# A systematic pan-cancer analysis identifies LDHA as a novel predictor for immunological, prognostic, and immunotherapy resistance

**DOI:** 10.18632/aging.205800

**Published:** 2024-05-03

**Authors:** Qiqi Zhang, Yuanning Luo, Bingshuo Qian, Xiuhua Cao, Caijun Xu, Kan Guo, Runlan Wan, Yaling Jiang, Tiecheng Wang, Zhiqiang Mei, Jinbiao Liu, Chaoxiang Lv

**Affiliations:** 1 The Research Center for Preclinical Medicine, Southwest Medical University, Luzhou, P.R. China; 2 Southwest Medical University, Luzhou, P.R. China; 3 Department of Oncology, The Affiliated Hospital of Southwest Medical University, Luzhou, P.R. China; 4 Changchun Veterinary Research Institute, Chinese Academy of Agricultural Sciences, Changchun, P.R. China; 5 School of Pharmacy, Henan University, Kaifeng, P.R. China; 6 Department of Gastroenterology, The Affiliated Hospital of Southwest Medical University, Luzhou, P.R. China; 7 Jiangsu Co-innovation Center for Prevention and Control of Important Animal Infectious Diseases and Zoonoses, Yangzhou University, Yangzhou, P.R. China

**Keywords:** LDHA, pan-cancer, single-cell RNA-Seq, tumor microenvironment, immunotherapy

## Abstract

Lactate dehydrogenase A (LDHA), a critical enzyme involved in glycolysis, is broadly involved multiple biological functions in human cancers. It is reported that *LDHA* can impact tumor immune surveillance and induce the transformation of tumor-associated macrophages, highlighting its unnoticed function of *LDHA* in immune system. However, in human cancers, the role of *LDHA* in prognosis and immunotherapy hasn’t been investigated. In this study, we analyzed the expression pattern and prognostic value of *LDHA* in pan-cancer and explored its association between tumor microenvironment (TME), immune infiltration subtype, stemness scores, tumor mutation burden (TMB), and immunotherapy resistance. We found that *LDHA* expression is tumor heterogeneous and that its high expression is associated with poor prognosis in multiple human cancers. In addition, *LDHA* expression was positively correlated with the presence of mononuclear/macrophage cells, and also promoted the infiltration of a range of immune cells. Genomic alteration of *LDHA* was common in different types of cancer, while with prognostic value in pan-cancers. Pan-cancer analysis revealed that the significant correlations existed between *LDHA* expression and tumor microenvironment (including stromal cells and immune cells) as well as stemness scores (DNAss and RNAss) across cancer types. Drug sensitivity analysis also revealed that *LDHA* was able to predict response to chemotherapy and immunotherapy. Furthermore, it was confirmed that knockdown of *LDHA* reduced proliferation and migration ability of lung cancer cells. Taken together, *LDHA* could serve as a prognostic biomarker and a potential immunotherapy marker.

## INTRODUCTION

Cancer is a leading cause of death in the world and has an important burden on the quality of life for patients [1–3]. Unluckily, the morbidity and mortality of cancer are increasing rapidly in most countries including North America and Europe [4, 5], and the current medical conditions cannot fully cure it yet. By 2020, the number of new cases and deaths from cancer worldwide will respectively increase to 19.3 million and 10 million [6]. The cases of global cancer are expected to reach 28.4 million in 2040, an increase of 47% over 2020 [7, 8]. As a result, cancer has become a growing burden on the whole society.

In contrast to well-differentiated normal cells that rely on pyruvate oxidation to produce energy for physiological function, tumor cells that rapidly proliferate rely on glycolysis for energy, a metabolic reprogramming known as the Warburg effect [9–12]. Even if there is enough oxygen, glycolysis can quickly provide energy supply for tumor cells and macromolecules, which forms an acidic environment that facilitates tumor cell metastasis [13–15]. Lactate dehydrogenase A (LDHA) is a critical enzyme involved in the glycolysis that converts pyruvate to lactic acid [16]. *LDHA* has diversified biological roles in cancer progression, such as inducing proliferation, promoting invasion, resulting in tumor metastasis, etc. [17–19]. Additionally, it was able to reduce the pH through lactic acid enrichment, which impacted tumor immune surveillance [20]. Recent studies have shown that LDHA drives the recruitment of immunosuppressive cells and induces the transformation of tumor-associated macrophages [21, 22], highlighting its unnoticed function in the immune system. Therefore, further study of the biological functions of LDHA and exploration of its potential molecular functions in cancer genesis and progression will help to deepen the understanding of tumors at the molecular level and provide an effective theoretical basis for the future study of new tumor therapeutic approaches.

Pan-cancer research facilitates a multifaceted understanding of the molecular variability in different cancers and is of great significance in identifying biomarkers for early cancer detection and targeted therapy [23–25]. Based on the databases such as TCGA (The Cancer Genome Atlas), multiple studies have analyzed thirty-three common tumors through multi-omics methods, providing unprecedented opportunities to discover the molecular mechanisms of different cancer types [26]. On account of the rapid progress in bioinformatics technology, the identification of cancer biomolecular markers and their functions have become a hot topic in current research [27]. This article focuses on identifying whether *LDHA* can be a potential marker of cancer, which is important for future clinical treatment of tumors and efficacy monitoring.

In our study, we analyzed the link of *LDHA* expression with tumor prognosis or the immune system based on a series of databases. Furthermore, we also revealed an association of *LDHA* expression with chemotherapy drug sensitivity in pan-cancer. Our study highlights the impact of LDHA expression on different aspects of pan-cancer in humans, providing possible theoretical knowledge for the effective treatment of malignant tumors, and LDHA has the potential to be used as a marker for cancer therapy.

## RESULTS

### The expression pattern of *LDHA* across human cancers

**Figure 1:**
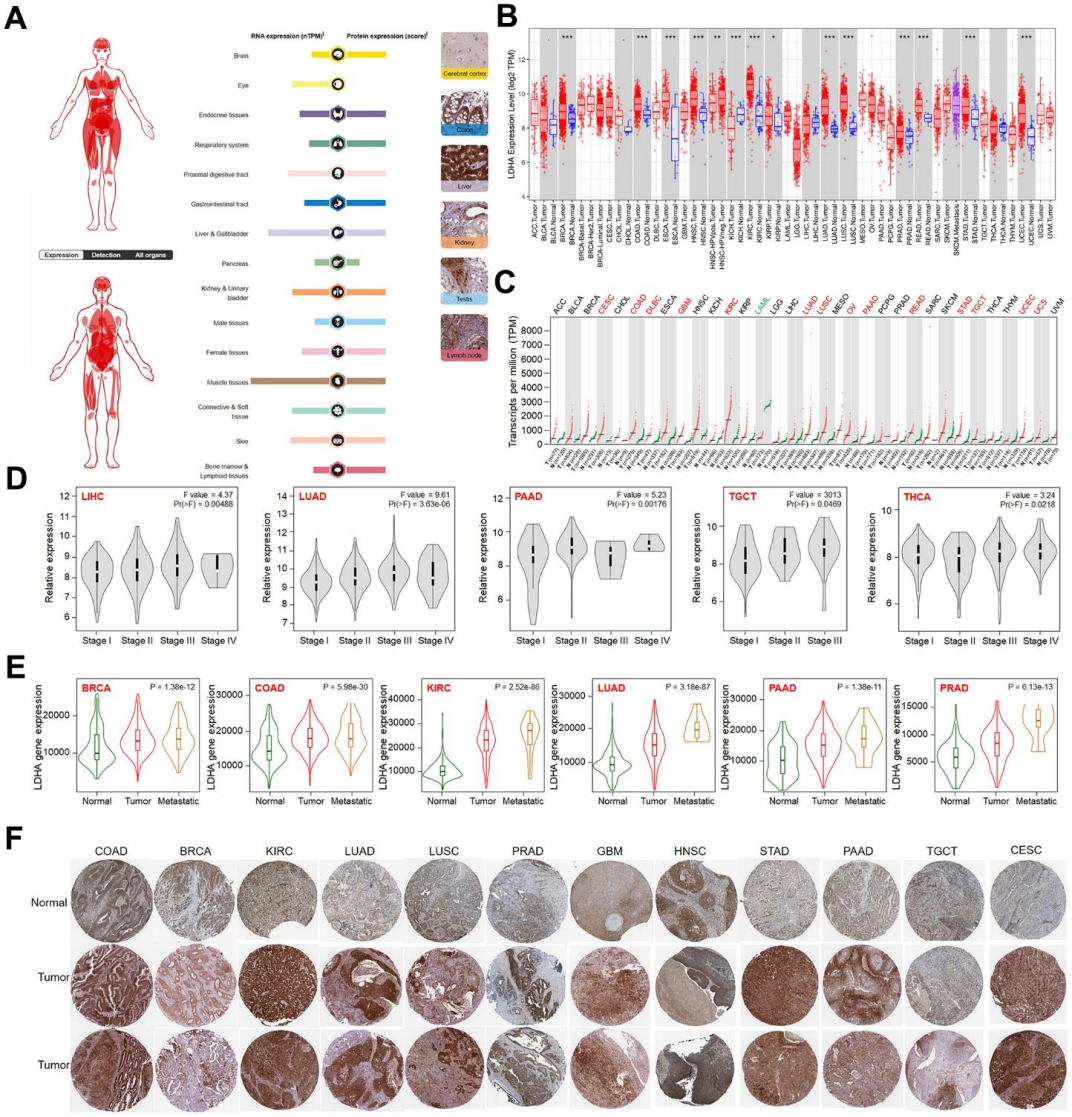
**Expression pattern of *LDHA* in pan-cancer.** (**A**) Summary of mRNA and protein expression. (**B**) The expression level of *LDHA* in tumor and normal tissues via TIMER 2.0. (**C**) *LDHA* expression status in different types of cancers from TCGA and GTEx data. (**D**) The expression of *LDHA* in different types of cancers (LIHC, LUAD, PAAD, TGCT, THCA) was analyzed according to pathological stage (stage I, stage II, stage III, and stage IV). (**E**) Differences in *LDHA* expression between normal, tumor, and metastatic tissues among BRCA, COAD, KIRC, LUAD, PAAD, and PRAD. (**F**) Based on the HPA (https://www.proteinatlas.org/) database, representative immunohistochemical staining of LDHA in normal and tumor tissues of different cancer types. The p < 0.05 was considered to be statistically significant. *p < 0.05, **p < 0.01, ***p < 0.001.

Initially, we analyzed the expression pattern of *LDHA* in various tissues. We observed that *LDHA* is expressed in brain, respiratory system, endocrine tissue, proximal digestive tract, gastrointestinal tract, liver, pancreas, gallbladder, bladder, kidney, male tissue (mainly in prostate and seminal vesicle), female tissue (mainly in uterus), muscle, soft tissue, skin, and lymphatic tissue (Figure 1A). Combining the data from TCGA database, we compared *LDHA* expression in cancerous tissue of thirty-three tumor types, the full name and abbreviation of tumor were shown in Table 1. *LDHA* is widely overexpressed in BRCA, COAD, ESCA, HNSC, KIRC, KIRP, LUAD, LUSC, PRAD, READ, STAD, and UCEC (Figure 1B). Considering the lack of corresponding normal controls for some tumors in the TCGA database, we analyzed the *LDHA* expression in TCGA and GTEx (Genotype-Tissue Expression) database in depth. The results suggested that *LDHA* expression was also significantly up-regulated in CESC, COAD, DLBC, GBM, KIRC, LUAD, LUSC, OV, PAAD, READ, STAD, TGCT, UCEC and UCS (Figure 1C). Additionally, we found a significant correlation between the expression of *LDHA* and the pathological stage of patients (Figure 1D), and its expression level was higher in metastatic tumors than in primary tumors, such as in BRCA, COAD, KIRC, LUAD, PAAD, and PRAD (Figure 1E). Using the HPA (https://www.proteinatlas.org/) database, we analyzed the protein expression in human tissues and found that LDHA was more powerfully expressed in tumor tissues than in adjacent normal tissues, images available from online-database (https://www.proteinatlas.org/search/LDHA) (Figure 1F). This finding is in agreement with data from the TCGA and GTEx database. LDHA protein expression was higher in COAD, BRCA, KIRC, LUAD, LUSC, PRAD, GBM, HNSC, STAD, PAAD, TGCT and CESC tumor tissues than in their adjacent normal tissues.

**Table 1:** Pan-cancer data acquired from TCGA database.

**Abbreviation**	**Full name**	**Tumor samples**	**Normal samples**
ACC	Adrenocortical carcinoma	79	0
BLCA	Bladder urothelial carcinoma	408	19
BRCA	Breast invasive carcinoma	1091	113
CESC	Cervical squamous cell carcinoma and endocervical adenocarcinoma	304	3
CHOL	Cholangiocarcinoma	36	9
COAD	Colon adenocarcinoma	456	41
DLBC	Lymphoid neoplasm diffuse large B-cell lymphoma		
ESCA	Esophageal carcinoma	161	11
GBM	Glioblastoma multiforme	161	5
HNSC	Head and neck squamous cell carcinoma	500	44
KICH	Kidney chromophobe	65	24
KIRC	Kidney renal clear cell carcinoma	534	72
KIRP	Kidney renal papillary cell carcinoma	288	32
LAML	Acute myeloid leukemia		
LGG	Brain lower grade glioma	511	0
LIHC	Liver hepatocellular carcinoma	371	50
LUAD	Lung adenocarcinoma	513	59
LUSC	Lung squamous cell carcinoma	501	49
MESO	Mesothelioma	86	0
OV	Ovarian serous cystadenocarcinoma	376	0
PAAD	Pancreatic adenocarcinoma	177	4
PCPG	Pheochromocytoma and paraganglioma	179	3
PRAD	Prostate adenocarcinoma	495	52
READ	Rectum adenocarcinoma	166	10
SARC	Sarcoma	259	2
SKCM	Skin cutaneous melanoma	468	1
STAD	Stomach adenocarcinoma	375	32
TGCT	Testicular germ cell tumors	150	0
THCA	Thyroid carcinoma	502	58
THYM	Thymoma	119	2
UCEC	Uterine corpus endometrial carcinoma	543	35
UCS	Uterine carcinosarcoma	56	0
UVM	Uveal melanoma	80	0

### Prognostic value of *LDHA* expression in pan-cancer data

**Figure 2:**
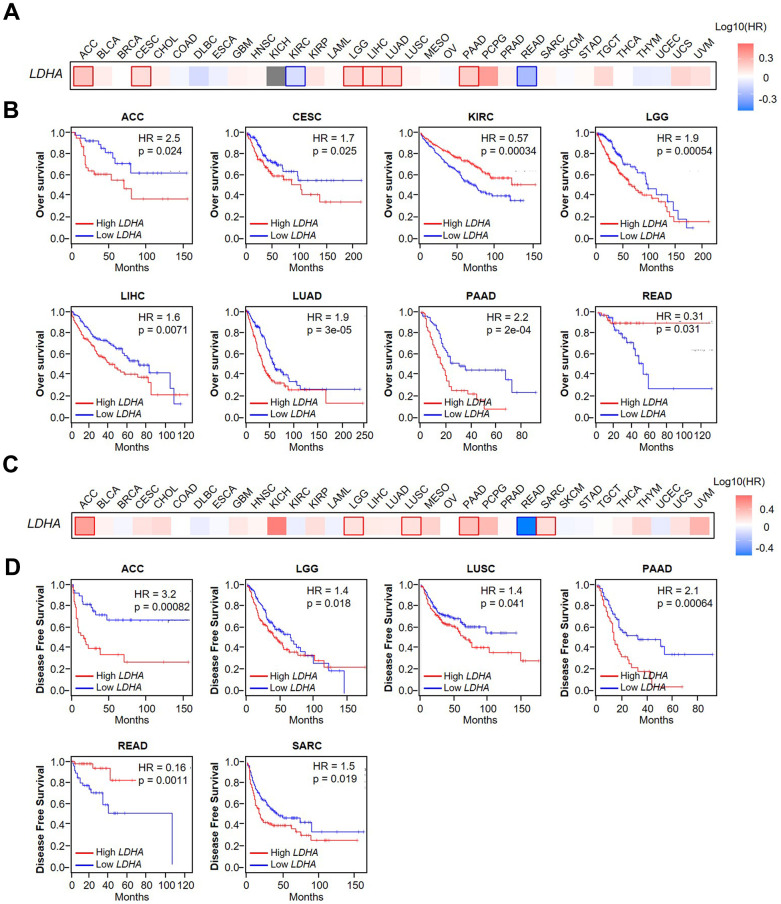
**Correlation between *LDHA* expression and overall survival and disease-free survival in patients with different cancer types.** (**A**) Using GEPIA2 to construct an overall survival (OS) map of *LDHA* expression. (**B**) Kaplan–Meier survival curves (OS) of patients with high or low *LDHA* expression in multiple human cancers (ACC, CESC, KIRC, LGG, LIHC, LUAD, PAAD, READ). (**C**) Using GEPIA2 to construct a disease-free survival (DFS) map of *LDHA* expression. (**D**) Kaplan–Meier survival curves (DFS) of patients with high or low *LDHA* expression in multiple human cancers (ACC, LGG, LUSC, PAAD, READ, SARC). The expression of *LDHA* was distinguished by different color lines. Red lines indicated high expression, and blue lines indicated low expression.

**Figure 3:**
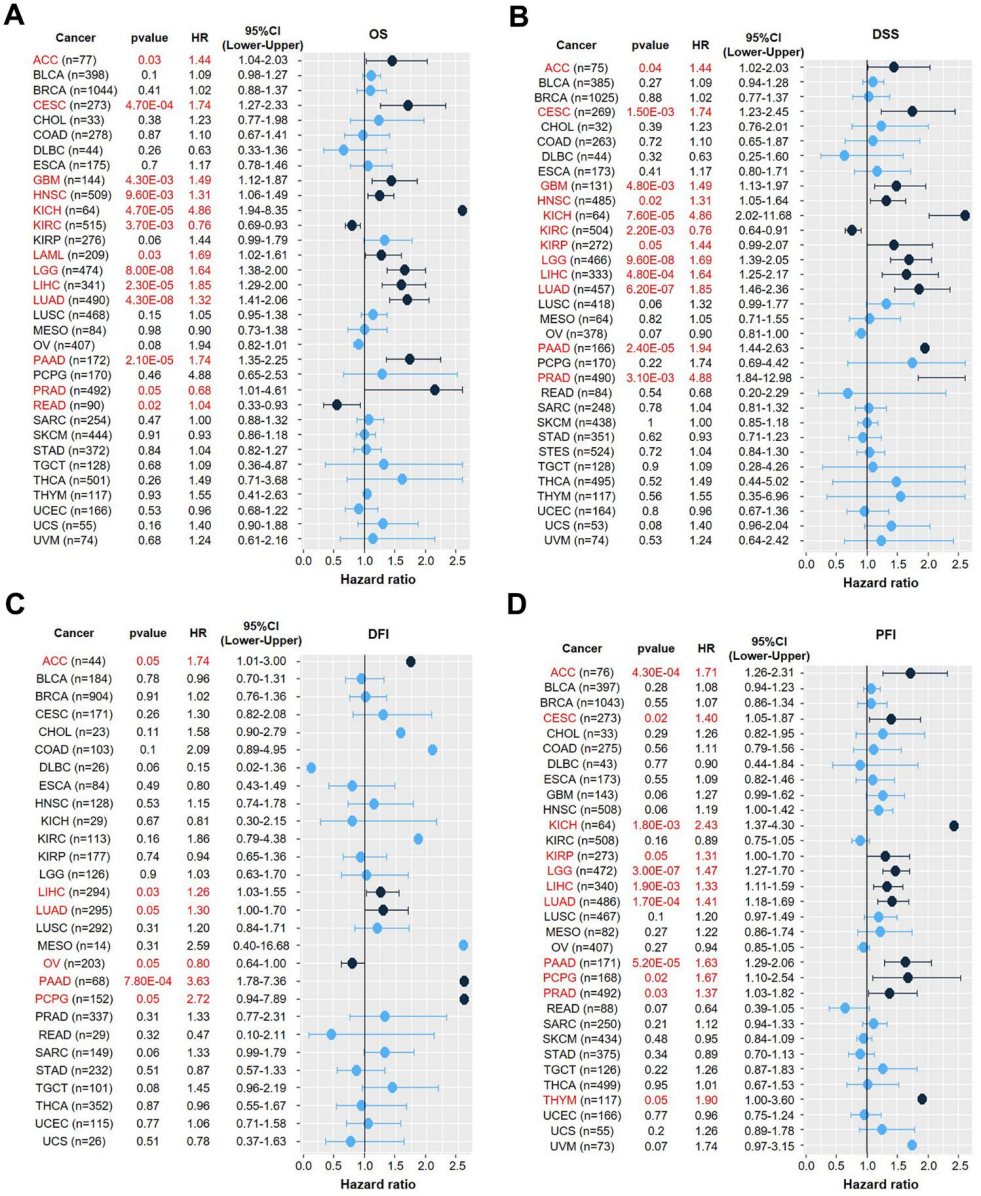
**Univariate Cox regression analysis of the correlation between *LDHA* expression and prognosis in 33 different tumors.** (**A**) Univariate Cox regression analysis of the correlation between *LDHA* expression and OS in different cancer types. (**B**) Univariate Cox regression analysis of the correlation of *LDHA* expression with DSS in different cancer types. (**C**) Univariate Cox regression analysis of the correlation between *LDHA* expression and DFI in different cancer types. (**D**) Univariate Cox regression analysis of the correlation of *LDHA* expression with PFI in different cancer types. The red words and black dots indicate that the gene is a risk factor in the corresponding tumor.

After that, we accessed the prognostic significance of *LDHA* expression in overall survival for pan-cancer in thirty-three cancer types (Figure 2A). We found that in ACC (p=0.024), CESC (p=0.025), LGG (p=0.00054), LIHC (p=0.0071), LUAD (p=3e-05), and PAAD (p=2e-04), the expression of LDHA was higher with poorer OS (Figure 2B). Besides, we also accessed the prognostic significance of *LDHA* expression in disease-free survival (DFS) for pan-cancer in thirty-three cancer types (Figure 2C). The results showed that higher *LDHA* expression was correlated with a shorter DFS time in ACC (p=0.00082), LGG (p=0.018), LUSC (p=0.041), PAAD (p=0.00064) and SARC (p=0.019) (Figure 2D). To further evaluate the association between *LDHA* expression and cancer prognosis, univariate Cox regression analysis was performed. Based on the results, it was concluded that *LDHA* expression was associated with the prognosis of a variety of tumors, including overall survival (OS) (Figure 3A), disease-specific survival (DSS) (Figure 3B), disease-free interval (DFI) (Figure 3C), and progression-free interval (PFI) (Figure 3D). Simultaneously, we also explored the correlation between differential expression of LDHA and various tumor outcomes. The analysis concluded that *LDHA* was a detrimental prognostic (OS, DSS, DFI, and PFI) factor in ACC, LIHC, LUAD, and PAAD (Table 2).

**Table 2:** The association between high expression of LDHA and prognosis of pan-cancer in TCGA database.

**Role**	**OS**	**DSS**	**DFI**	**PFI**
**Protective**	KIRC, PRAD	KIRC	OV	
**Detrimental**	**ACC**, CESC, GBM, HNSC, KICH, LAML, LGG, **LIHC**, **LUAD**, **PAAD**, READ	**ACC**, CESC, GBM, HNSC, KICH, KIRP, LGG, **LIHC**, **LUAD**, **PAAD**, PRAD	**ACC**, **LIHC**, **LUAD**, **PAAD**, PCPG	**ACC**, CESC, KICH, KIRP, LGG, **LIHC**, **LUAD**, **PAAD**, PCPG, PRAD, THYM

Annotation: OS, overall survival; DSS, Disease special survival; DFI, Disease free interval; PFI, Progression free interval.

### The links of *LDHA* expression to immune cell infiltration

**Figure 4:**
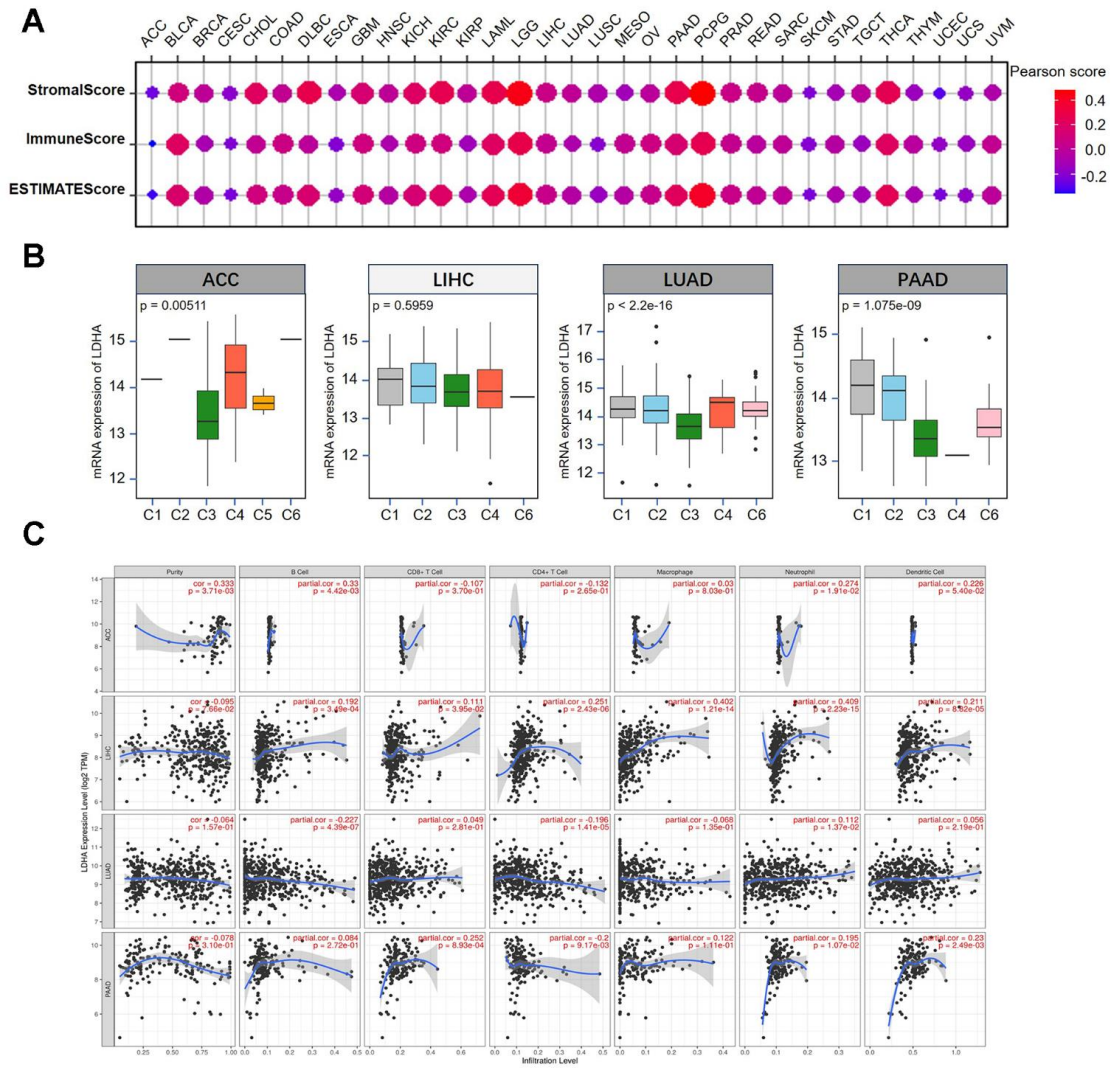
**Correlation between *LDHA* expression and tumor microenvironment in different types of cancers.** (**A**) The correlation of *LDHA* expression with stromal score, immune score and ESTIMATE score. (**B**) The relationship between *LDHA* expression and immune infiltration subtypes multiple human cancers (ACC, LIHC, LUAD, PAAD). (**C**) The correlations of *LDHA* expression with immune cell infiltration in ACC, LIHC, LUAD, and PAAD.

Stromal cells, fibroblasts and immune cells play important roles in regulating the TME during tumor progression [28]. To further explore the relationship between *LDHA* expression and TME, we utilized the ESTIMATE algorithm that was presented as a matrix and immune score. Statistical data showed that *LDHA* expression was significantly positively or negatively correlated with stromal score, immune score and ESTIMATE score (Figure 4A). Additionally, we observed a significant correlation of *LDHA* expression with the immune infiltration subtypes of ACC (p=0.00511), LUAD (p<2.2e-16), and PAAD (p=1.075e-09) (Figure 4B).

**Figure 5:**
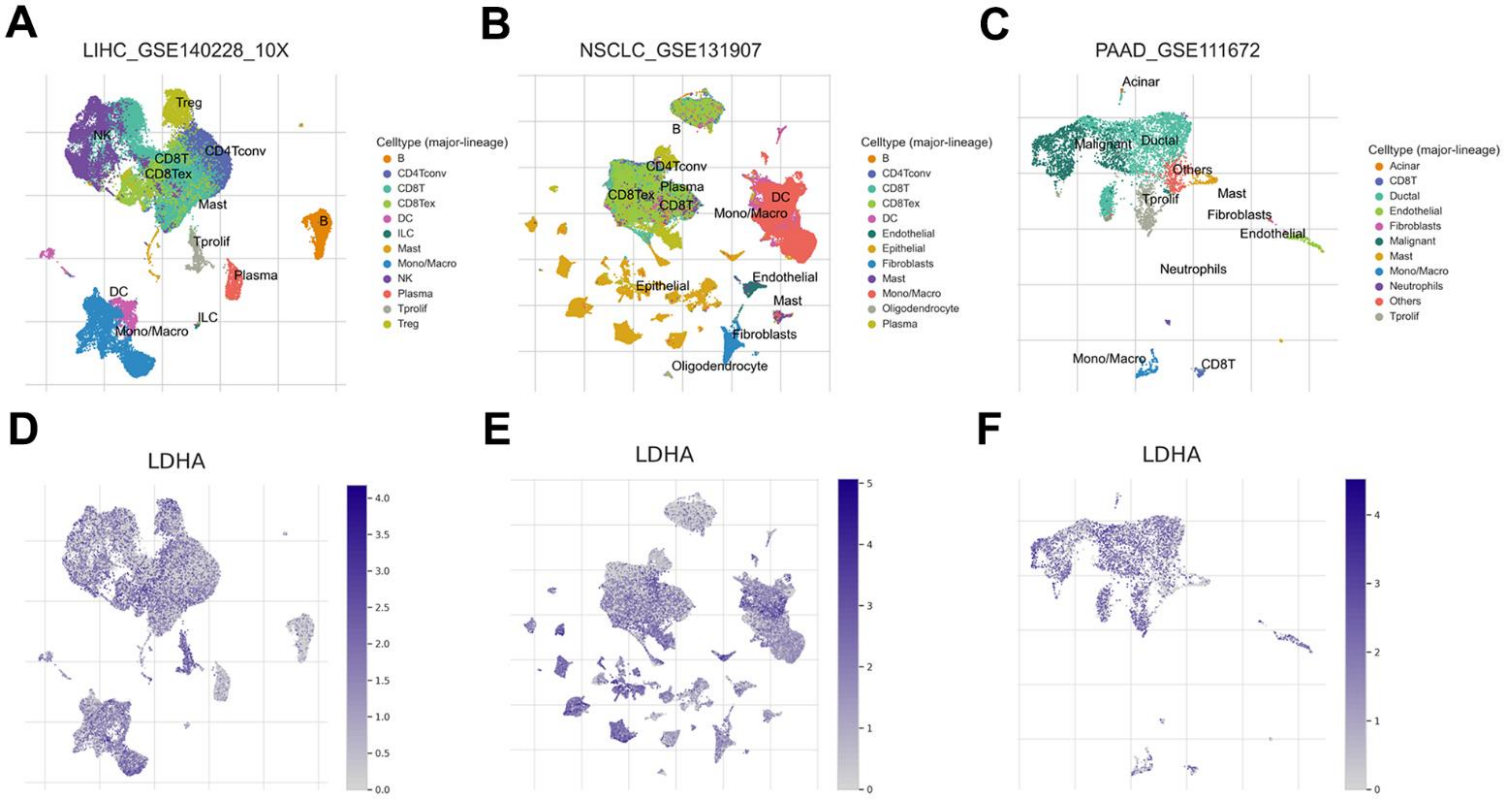
***LDHA* expression at the single-cell level.** (**A**–**C**) UMAP plots showing cell clusters in different cell types of multiple cancers, including LIHC, NSCLC, and PAAD. (**D**–**F**) The expression levels of *LDHA* in different cell types in different types of human cancers, including LIHC, NSCLC, and PAAD.

Furthermore, we investigated the correlation between immune cell infiltration and *LDHA* expression. As shown in Figure 4C, *LDHA* expression had a positive correlation with tumor purity, B cells and neutrophils in ACC. In LIHC, *LDHA* expression was positively linked to the presence of B cells, CD8^+^ T cells, CD4^+^ T cells, macrophages, neutrophils, and dendritic cells. When analyzing immune cell infiltration, we also found that LDHA expression was positively related to the presence of B-cells, CD4^+^ T cells and neutrophils in LUAD. Additionally, we also found that *LDHA* expression has a positive correlation with CD8^+^ T cells, CD4^+^ T cells, neutrophils, and dendritic cells in PAAD. To investigate the potential mechanism of *LDHA* affecting TME, we refined single-cell RNA-seq (scRNA) sequencing to explore its response in various immune cells. The scRNA datasets were obtained from public online database (http://tisch1.comp-genomics.org), including LIHC (Figure 5A), NSCLC (Figure 5B), and PAAD (Figure 5C). Our analysis showed that its expression is mainly in monocytes/macrophages, especially in LIHC (Figure 5D), NSCLC (non-small cell lung cancer) (Figure 5E), and PAAD (Figure 5F).

**Figure 6:**
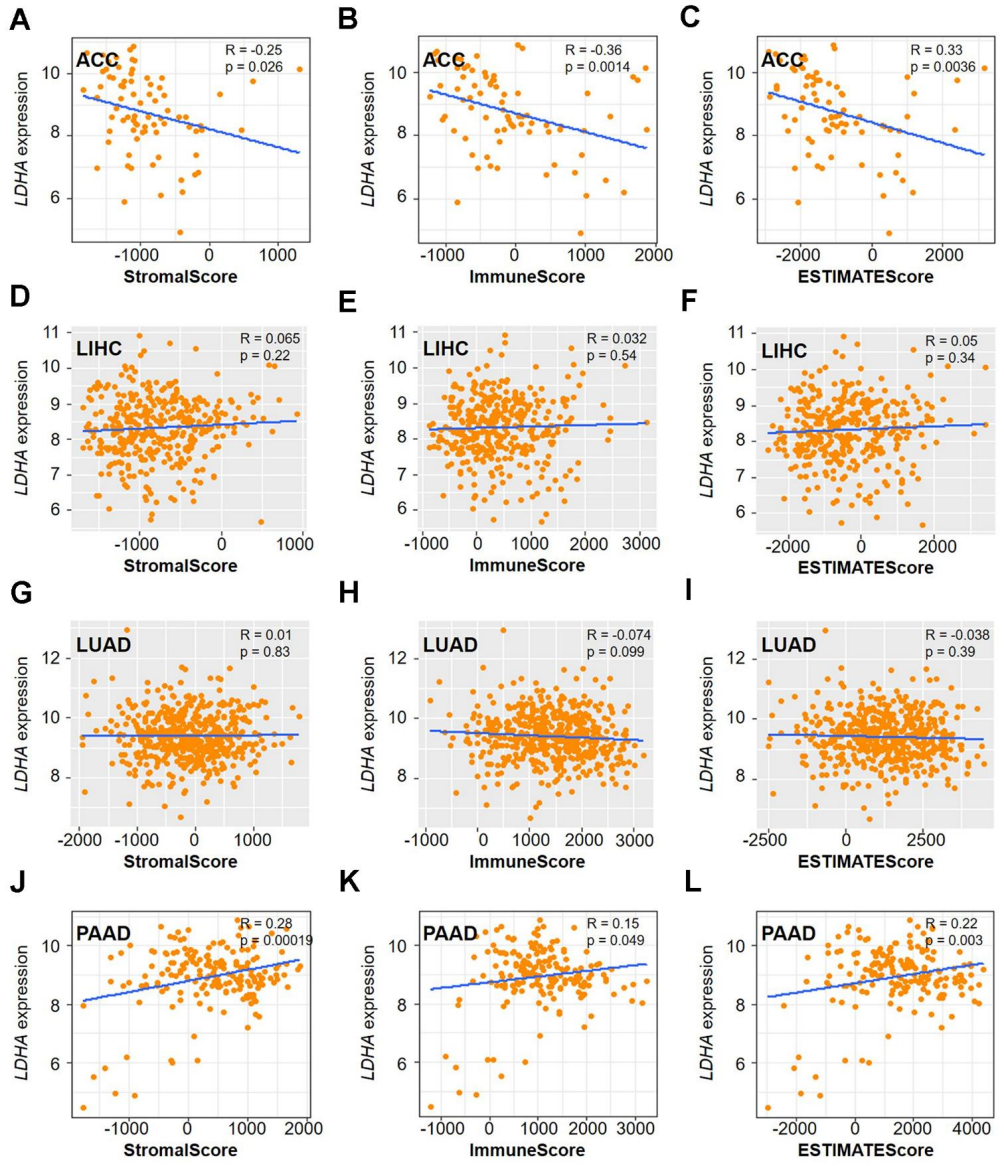
**Correlation analysis of *LDHA* expression with TME in multiple cancers.** The correlation of *LDHA* expression with stromal score, immune score, and ESTIMATE score in ACC (**A**–**C**), LIHC (**D**–**F**), LUAD (**G**–**I**), and PAAD (**J**–**L**). Gray brown background indicates no significant correlation between the gene expression and the corresponding index (p >0.05). Light background indicates that the gene is significantly correlated with the corresponding index (p <0.05). R represents correlation value, R >0 means positive correlation, R <0 means negative correlation.

We previously showed that *LDHA* was a detrimental prognostic (OS, DSS, DFI, and PFI) factor in ACC, LIHC, LUAD, and PAAD. Subsequently, we concentrate on the correlation between *LDHA* expression and immune invasion in these 4 tumors (Figure 6). Based on the results, it can be concluded that *LDHA* expression had a significant negative correlation with stromal score (Figure 6A), immune score (Figure 6B) and ESTIMATE score (Figure 6C) in ACC. However, we did not observe a similar association in LIHC (Figure 6D–6F) and LUAD (Figure 6G–6I). In contrast, *LDHA* expression has a positive association with the stromal score (Figure 6J), immune scores (Figure 6K) and ESTIMATE score (Figure 6L) in PAAD. Taken together, these findings suggest that *LDHA* is strongly associated with tumor immune cell infiltration.

### The correlation of *LDHA* expression with stemness score

**Figure 7:**
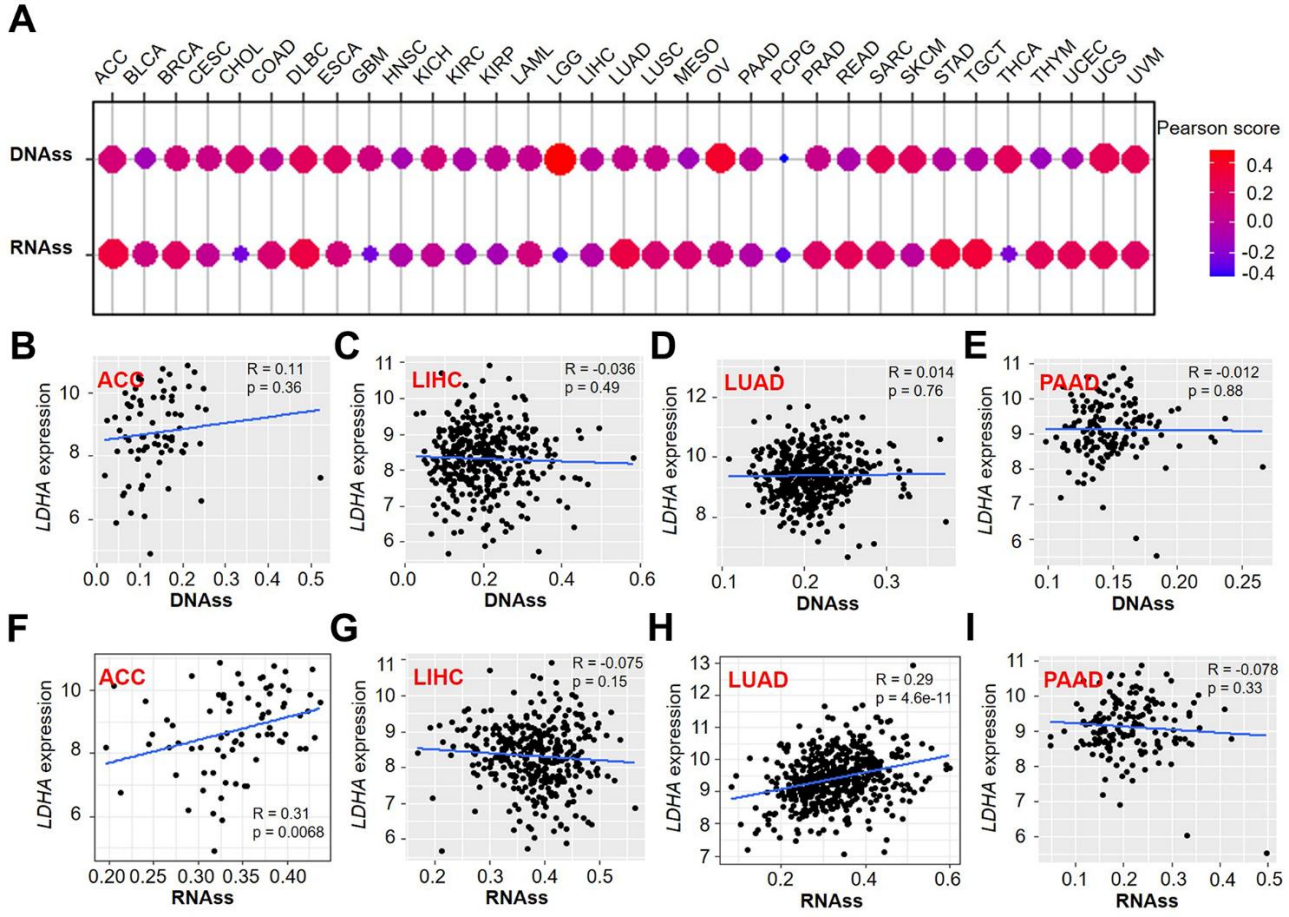
**Association of *LDHA* expression with stemness score in pan-cancer.** (**A**) The correlation of *LDHA* expression with DNAss and RNAss. (**B**–**E**) *LDHA* expression correlated with DNAss in ACC, LIHC, LUAD, and PAAD. (**F**–**I**) The expression of *LDHA* correlated with RNAss in ACC, LIHC, LUAD, and PAAD. Gray brown background indicates no significance between the gene expression and the corresponding index (p >0.05). Light background indicates that the gene is significantly correlated with the corresponding index (p <0.05). R represents correlation value, R >0 means positive correlation, R <0 means negative correlation.

The previous results suggested *LDHA* expression is relevant to prognosis and immune cell infiltration in some human cancers. To further analyze the biological roles of *LDHA* expression in tumor stemness, the correlation analysis was performed. We observed that *LDHA* expression was positively or negatively correlated with DNAss and RNAss (Figure 7A). Similarly, we further investigated the hidden relationship among *LDHA* expression and stemness score in ACC, LIHC, LUAD, and PAAD (Figure 7B–7E). We observed that *LDHA* expression was remarkably positively correlated with RNAss in ACC (Figure 7F) and LUAD (Figure 7H). These findings mean that *LDHA* is strongly related to tumor stemness, especially in ACC and LUAD.

### Effect of *LDHA* expression on cancer chemotherapy sensitivity

**Figure 8:**
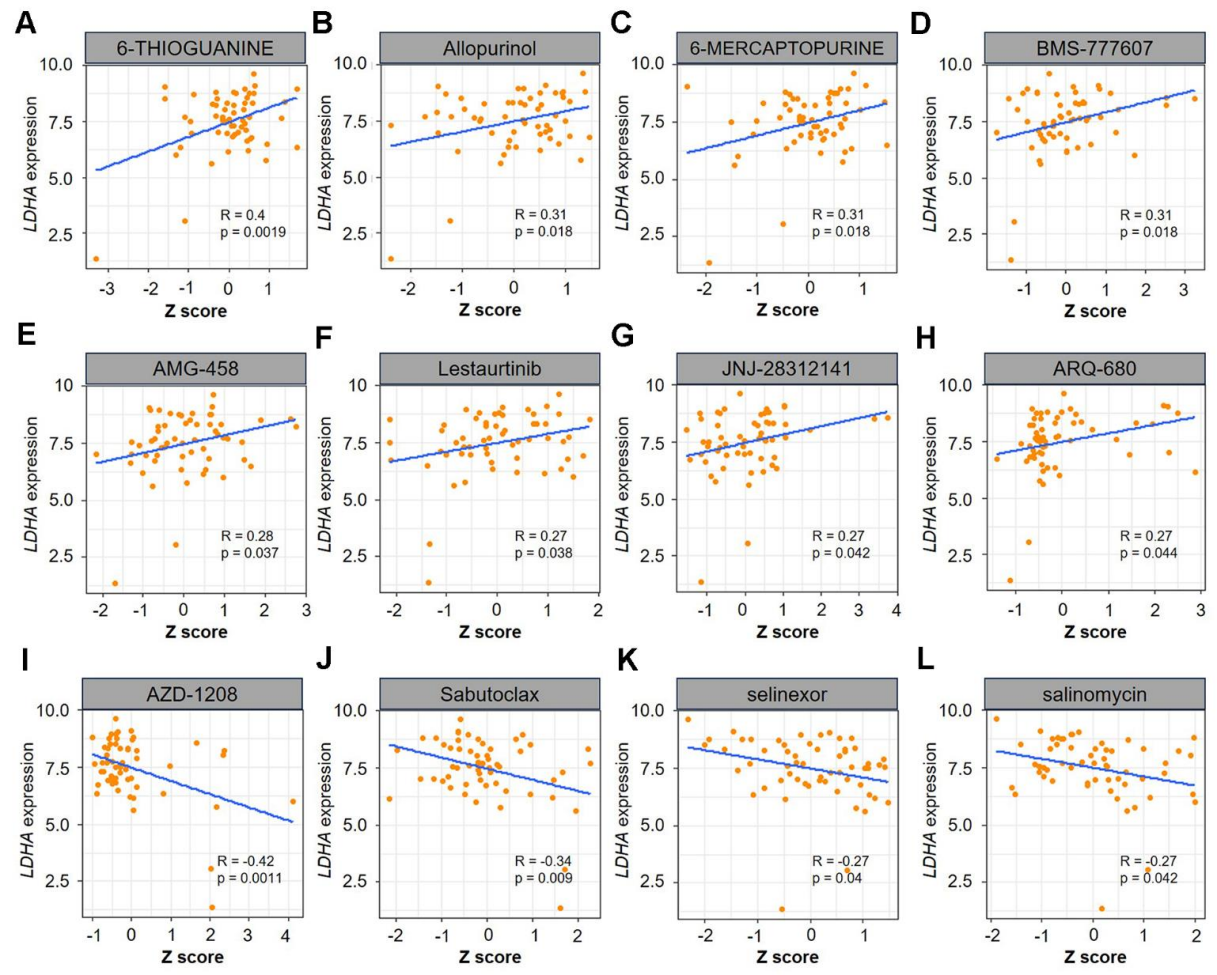
**Relationship between *LDHA* expression and drug sensitivity.** *LDHA* expression was positively correlated with drug sensitivity of 6-THIOGUANINE (**A**), Allopurinol (**B**), 6-MERCAPTOPURINE (**C**), BMS-777607 (**D**), AMG-458 (**E**), Lestaurtinib (**F**), JNJ-28312141 (**G**), and ARQ-680 (**H**), but negatively correlated with the drug sensitivity of AZD-1208 (**I**), Sabutoclax (**J**), Selinexor (**K**), Salinomycin (**L**). R represents correlation value, R >0 means positive correlation, R <0 means negative correlation. The p < 0.05 was considered to be statistically significant.

Improving drug sensitivity is not only a major strategy for cancer treatment, but also an essential option to prevent tumor cells from developing drug tolerance [29]. Therefore, we further analyzed that the correlation of *LDHA* expression with the sensitivity of 60 human cancer cell lines (NCI-60) to more than 200 chemotherapeutic drugs. The results showed that *LDHA* expression was significantly positively correlated with the drug sensitivity of 6-THIOGUANINE, Allopurinol, 6-MERCAPTOPURINE, BMS-777607, AMG-458, Lestaurtinib, JNJ-28312141, and ARQ-680 (Figure 8A–8H). Conversely, *LDHA* expression had a significant negative correlation with drug sensitivity of AZD-1208, Sabutoclax, Selinexor and Salinomycin (Figure 8I–8L). These findings indicate that *LDHA* expression is strongly related to the sensitivity of some chemotherapy drugs, which has significant implications for cancer therapeutic research targeting the LDHA gene in the future.

### *LDHA* genetic alterations across cancers

**Figure 9:**
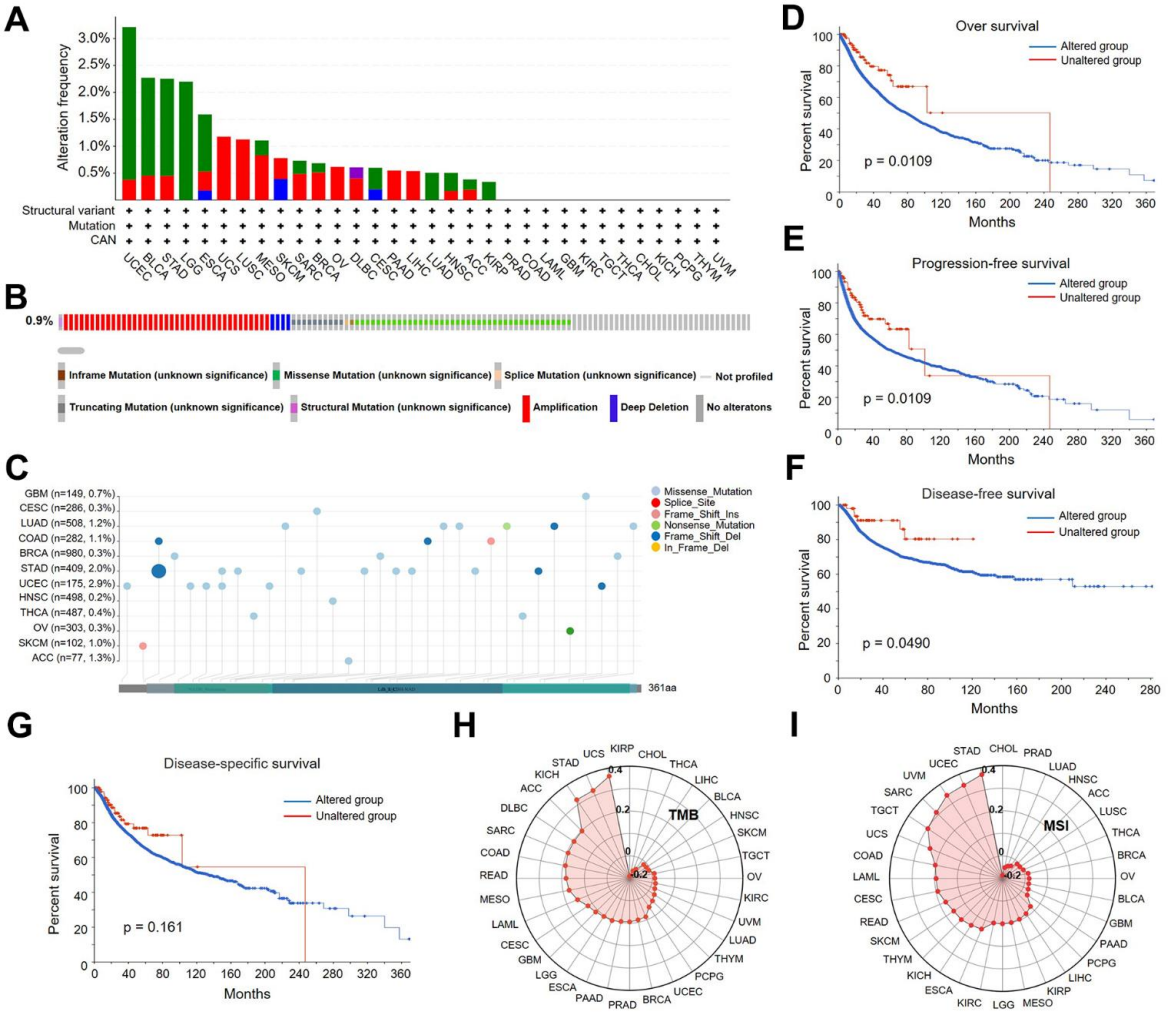
**Analysis of *LDHA* genetic alterations in different types of cancers.** (**A**) The frequency of *LDHA* mutations with mutation type across TCGA cancers by cBioPortal. (**B**) Oncoprint of *LDHA* gene alterations in cancer cohorts. (**C**) Mutation sites of the *LDHA* in TCGA samples. (**D**–**G**) The associations of pan-cancer *LDHA* mutation status with OS, DSS, DFS and PFS by cBioPortal. (**H**) The association of *LDHA* expression with TMB in pan-cancer. (**I**) The association of *LDHA* expression with MSI in pan-cancer.

Using the cBioPortal database, we attempted to study the genetic alterations of LDHA in a wide range of cancers. The results showed that *LDHA* amplification patterns exhibited in most cancer types, particularly in UCEC (Figure 9A). In addition, we found missense mutations, amplifications and deep deletions to be the major mutations in the LDHA gene (Figure 9B). We also presented the integrated data on mutations with important domains of *LDHA* in different cancer contexts, which are the higher frequency (Figure 9C). Based on the above, we further analyzed whether the alteration of *LDHA* affected the prognosis of different malignant tumors. Our analysis showed that in pan-cancer, *LDHA* mutation status was significantly correlated with OS (Figure 9D), PFS (Figure 9E), and DFS (Figure 9F), but not DSS (Figure 9G). These findings indicate that *LDHA* genetic alteration is related to poor prognosis in human cancers.

Tumor mutational burden (TMB), neoantigens, and microsatellite instability (MSI) in the TME are closely associated with anti-tumor immunity and may predict the effectiveness of cancer immunotherapy [30]. Consequently, we discussed the association of *LDHA* expression with TMB and MSI in pan-cancer. The findings suggested that the expression of *LDHA* was strongly positively correlated with the TMB of UCS, STAD, and KICH, but negatively correlated with KIRP and CHOL (Figure 9H). Additionally, we observed that *LDHA* expression had a positive correlation with the MSI of STAD, UCEC, UVM, SARC, and TGCT, but negatively in CHOL (Figure 9I). Combing with the previous findings, we concluded that *LDHA* may play a role in monitoring anti-tumor immunotherapy by modulating TME composition and immune responses.

### Effect of *LDHA* on epithelial-mesenchymal transition (EMT) in lung cancer

**Figure 10:**
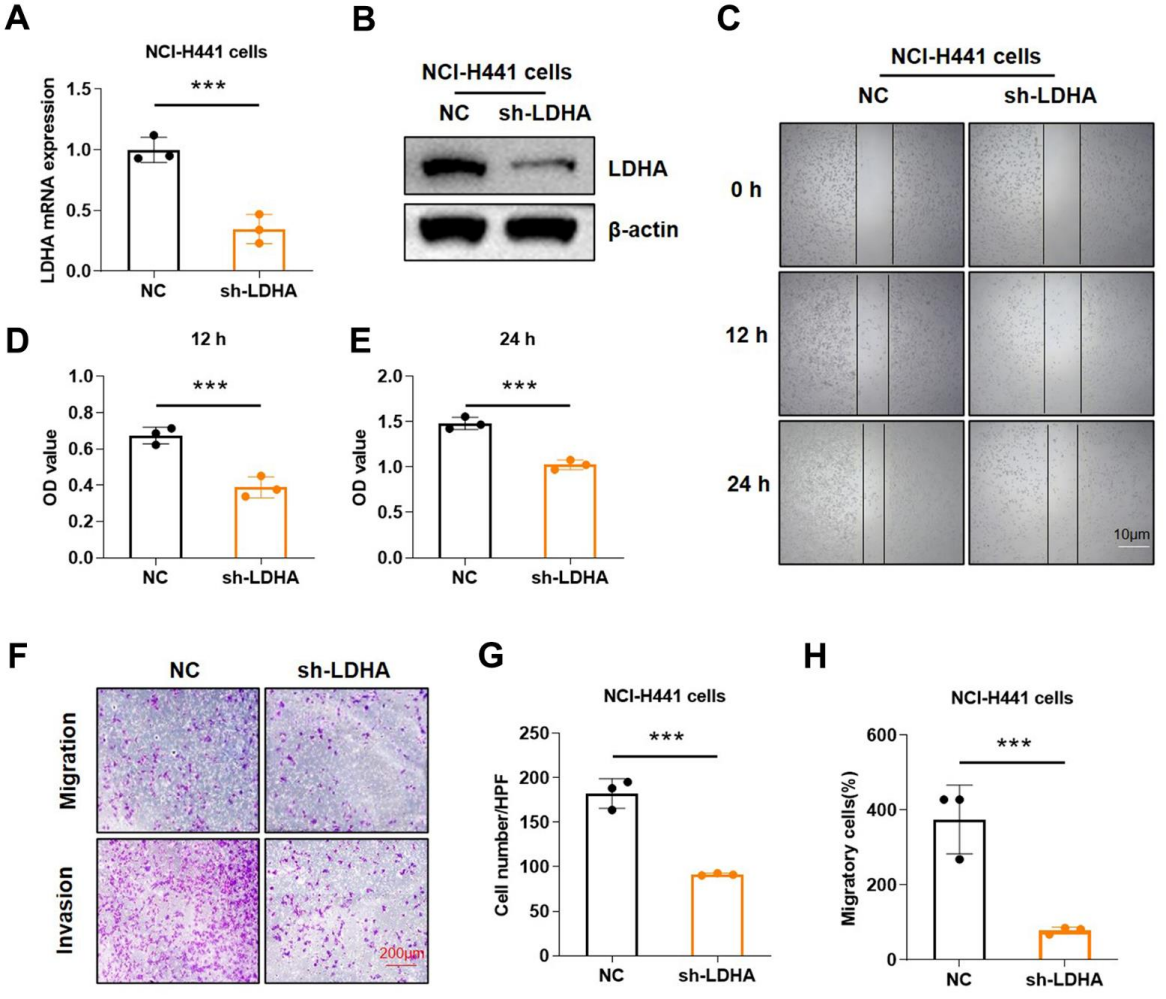
**Knockdown of *LDHA* inhibited proliferation and migration of LUAD.** (**A**, **B**) Knockdown of *LDHA* was verified by RT-qPCR and western blot in NCI-H441 cells. (**C**) Microscopic observations were recorded at 0, 12, and 24 h after scratching the surface of a confluent layer of the indicated NCI-H441 cells. (**D**, **E**) MTT assay suggested that knockdown of *LDHA* inhibited the proliferation of NCI-H441 cells. (**F**) The effects of *LDHA* on cell migration and invasion were examined by transwell assays in NCI-H441 cells. (**G**) Quantitative analysis of cell migration in NCI-H441 cells. (**H**) Quantitative analysis of cell invasion in NCI-H441 cells. *p < 0.05, **p < 0.01, ***p < 0.001.

After a series of data analysis, we initially grasped the significance of different expressions of LDHA on human tumors. To further confirm the biological role of LDHA, we chose the human lung adenocarcinoma cell line NCI-H441 to conduct the experiment. We primarily transfected shRNA plasmid targeting *LDHA* to knock down endogenous *LDHA* in cells (Figure 10A, 10B). The wound healing assay showed that down-regulation of *LDHA* significantly decreased cell migration (Figure 10C). We also found that *LDHA* knockdown inhibited NCI-H441 cell proliferation (Figure 10D, 10E). To further explore the effects of *LDHA* expression on cell invasion and migration, transwell assay was performed (Figure 10F). Quantitative analysis showed that down-regulation of LDHA repressed cell invasion (Figure 10G) and migration (Figure 10H). After comprehensive analysis, we concluded that knockdown of *LDHA* inhibited the invasion and migration of NCI-H441 cells.

## DISCUSSION

Here, we analyzed in depth the expression level of *LDHA* based on several databases and dissected the associations of its expression with prognosis, immune infiltration, TME and TMB. Additionally, we also evaluated the potential contribution of *LDHA* to drug resistance for immunotherapy of cancers. We revealed the association between *LDHA* expression and tumor metastasis. These findings mainly demonstrate the biological roles of *LDHA* in human tumors occurrence and development, inspiring a theoretical basis for further exploration of its function in cancer therapy.

As a critical enzyme of the Warburg effect, LDHA is conducive to the linkage of the various steps of glycolysis and accelerates the conversion of glucose to lactate [31]. Previous studies suggested that increased lactate secretion can affect enzymes and co-enzyme function, with negative effects on cell metabolism providing energy and materials for tumor cells [32]. In addition, *LDHA* promotes the proliferation of tumor cells by increasing lactic acid production [33]. Inhibition of *LDHA* expression induces apoptosis in a variety of tumor cells, such as breast, liver, gastric and colorectal cancers [34]. This implies that targeted disruption of LDHA and its associated signaling pathways may provide a pathway to inhibit tumor progression. According to current study, we reveled that *LDHA* expression were dramatically upregulated in 14 tumor tissues compared to normal tissues. This suggests that *LDHA* plays important roles in the occurrence and pullulate of tumors.

It has been proved that cancer cells can increase the aerobic glycolysis rate by highly expressing LDHA, so that they can still obtain sufficient energy supply for their survival even in the adverse environment of hypoxia, and create an acidic environment conducive to malignant cells, causing immunosuppression, which also promotes tumor resistance [35]. For example, a high glycolytic state is correlated with a poor prognosis in colorectal cancer patients treated with first-line chemotherapy plus bevacizumab [36]. On the contrary, targeted inhibition of METTL3/LDHA axis can improve the sensitivity of colorectal cancer cells to 5-FU *in vivo* and *in vitro* [37]. Patients with metastatic gastric glands that have high mRNA expression of LDHA and other core enzymes in their primary tumors after second-line paclitaxel-ramucirumab treatment, patients with high *LDHA* expression have lower overall survival and progression-free survival than patients with low expression [38]. Up-regulation of LDHA expression activates the glycolytic pathway and also enhances the resistance of breast cancer cells to aromatase agents [39]. Here, a multifaceted bioinformatics analysis was conducted to investigate the correlation of *LDHA* expression patterns in various cancers with the corresponding tumor prognosis and stage, and we reveled that the prospect of *LDHA* in cancer diagnosis is worthy of expectation. We also analyzed the potential correlation between *LDHA* expression and drug sensitivity in human cancer cell lines. Expression of *LDHA* was positively correlated with the drug sensitivity of 6-THIOGUANINE, Allopurinol, 6-MERCAPTOPURINE, BMS-777607, AMG-458, Lestaurtinib, JNJ-28312141, and ARQ-680, while negatively correlated with AZD-1208, Sabutoclax, Selinexor, Salinomycin. These findings and current results provide sufficient evidence that *LDHA* expression is strongly associated with sensitivity to multiple chemotherapy agents. This provides an important reference for the prognostic monitoring of cancer, and also makes a potential contribution to the future research on cancer treatment targeting *LDHA*.

The genetic characteristics and phenotypes of gene expression in pan-cancer tissues are closely related to the activation status of their specific signaling pathways [40]. Under some circumstances, tumors count on these properties for their invasion, infiltration and metastasis [41, 42]. In this study, we found that *LDHA* expression was correlated with cancer stage, metastasis, invasion, immune regulation, etc., suggesting that it may be an independent predictor of tumor. To validate the accuracy of the analysis, we improved the transwell assay and scratch test of *LDHA* in tumor cells, and found that high *LDHA* expression promoted the proliferation, migration and invasion of lung cancer cell line NCI-H441. Furthermore, we also found that *LDHA* was mutated in most types of tumors. Among them, UCEC and STED had the highest mutation rate, suggesting that we should pay more attention to the connection between LDHA gene mutations and their associated cancer advancement.

Briefly, we found significant variations in *LDHA* expression between tumor and para-cancerous by pan-cancer analysis, and revealed the relevance of its expression to clinical prognosis. *LDHA* expression showed tumor heterogeneity, and it has the potential to be an independent prognostic factor for many tumors. The expression of *LDHA* were positively correlated with immune-related expression levels in multiple type of tumors. Furthermore, we also revealed the link of *LDHA* expression with immune subtypes, tumor stemness and drug resistance. These findings will help to further elucidate the mechanism of action of *LDHA* in cancer progression and provide broad strategies for treatment options in cancer patients.

## MATERIALS AND METHODS

### Data mining

We mined the RNAseq (HTSeq-FPKM) datasets for thirty-three types of human cancer, as well as clinical data, relevant immune subtypes data and stemness score data from the TCGA database (https://portal.gdc.cancer.gov/). The main information on thirty-three types of human tumors is presented in Table 1. In summary, we also searched for immunohistochemical images of LDHA through the Human Protein Atlas database, image available from online database (https://www.proteinatlas.org/search/LDHA). R version 4.3.0 has been used for data analysis.

### Expression analysis of *LDHA* in pan-cancer

Using an online database (TIMER, http://timer.cistrome.org/), we compared the expression of *LDHA* in human pan-cancers. The expression pattern of *LDHA* was analyzed by using Wilcoxon rank-sum test, and visualized by R-packages “ggplot2”. GEPIA2 is a cancer data access website based primarily on the TCGA and GTEx programs [43]. Subsequently, we also elucidated the variation of *LDHA* in normal tissues and corresponding malignant tissues in patients with different cancer types, as well as determined the relevance of its expression with the pathological tumor stages based on the GEPIA2 database. Using TNM plotter (https://tnmplot.com/analysis/), we investigated the differences of *LDHA* expression in normal tissues, tumor tissues, and metastatic tissues.

### Survival prognosis and Cox regression analysis in pan-cancer

Using GEPIA2, we obtained OS data, DFS data and survival curves. Then, the median expression level of *LDHA* was opted for the intergroup threshold. The survival curve is compared with Log-rank, and the hazard ratio (HR) is displayed. A log rank p < 0.05 was recognized as statistically significant. The “COX Univariate Regression” module was used to study the association between gene expression and clinical prognosis in human malignant tumors, including OS, DSS, DFI, and PFI. Forest plots were constructed using the R package “forestplot” and the relationship between LDHA expression and survival of tumor patients was assessed using hazard ratios (HR), HR <1 represented low risk and HR >1 represented high risk. Log-rank p <0.05 was considered statistically significant.

### Tumor microenvironment correlation analysis

The “ESTIMATE algorithm” was performed to analyze the correlation between gene expression and TME in human cancers by calculating stromal score and immune score [44]. Then, we used Pearson correlation coefficient to determine the association between TME and genes expression. R-packages “cor. Test”, “ggpubr” and “limma” were performed to visualize the results analysis. R represented the correlation coefficient, with R > 0 indicating a positive correlation and R < 0 indicating a negative correlation. p < 0.05 is defined as statistically significant.

### Immune infiltration subtype and stemness score in pan-cancer

From the UCSC database, the immune subtypes datasets were downloaded to perform immune infiltration subtype analysis. It includes six different immune subtypes from C1 to C6 [45]. The R-packages “limma” and “reshape2” were used for data processing and analysis. The visual result presentation was performed using R-packages “ggplots”, and “Wilcox. test” was used for significant difference. Correlations between gene expression and stemness scores were revealed by using R-packages “cor. Test”, “ggpubr” and “limma”, including DNA stemness score (DNAss) and RNA stemness score (RNAss). R represented the correlation coefficient, R >0 is a positive correlation and R <0 is a negative correlation. p < 0.05 was recognized as statistically significant.

### Immune cell and single-cell sequencing analysis

Using the TIMER database (https://cistrome.shinyapps.io/timer/), we obtained the RNA-Seq expression profiling data to analyze immune cell infiltration in tumor tissues, including B cell, CD4^+^ T cell, CD8^+^ T cell, macrophages, neutrophils and dendritic cells. The relevance of gene expression with immune cell infiltration was performed in multiple cancers for analysis. The statistical significance of R and P was defined as described above. R represented the correlation coefficient, R >0 was positive correlation, R <0 was negative correlation. The p < 0.05 was considered to be statistically significant. For single-cell sequencing analysis, we used interactive single-cell transcriptome visualization to break down the association of gene expression with the TME via a combined web resource called the Single-Cell Center for Tumor Immunity (TISCH, http://tisch.comp-genomics.org/) [46]. Expression of *LDHA* at the single-cell level in the LIHC_GSE140228, NSCLC_GSE131907, and PAAD_GSE111672 datasets was visualized with the “dataset” module. The image available from the online website of the public database (http://tisch1.comp-genomics.org/gallery/?cancer=LIHC&cancer=PAAD&cancer=NSCLC&species=). The analysis of the target gene is performed in the “Compare” module.

### Drug sensitivity analysis

From the CellMiner™ database (http://discover.nci.nih.gov/cellminer), we obtained the RNAseq expression data in 60 different humanized cancer cell lines (NCI-60) and drug sensitivity data [47]. The relevance of gene expression to drug resistance was further explored using Pearson correlation analysis. The visual analysis was performed by using R-package “ggplot2”. R represented the correlation coefficient, R >0 respected positive correlation, R <0 respected negative correlation. The p < 0.05 was viewed as statistically significant.

### Genetic alteration analysis in pan-cancer

Considering the integration of genetic alterations data in pan-cancer, we explored LDHA changes in human cancer based on an online database (https://www.cbioportal.org/). The gene alteration landscape in cancer was observed through the “Cancer Type Summary” module, and then the generate site plot was generated in the “Mutation” module [48]. Patients were grouped according to whether *LDHA* was altered or not, and the survival curve was constructed through the “Compare/survival” module to probe into the relationship under *LDHA* and clinical outcomes of pan-cancer patients. The p < 0.05 was recognized as statistically significant.

### Cell culture, plasmids and transfection assay

The humanized lung adenocarcinoma cell lines NCI-H441 was purchased from the Changchun Veterinary Research Institute (Changchun, China). The cells were cultured in Roswell Park Memorial Institute 1640 (RPMI-1640) medium containing 10% fetal bovine serum (Gibco; USA) with 100 μg/mL penicillin and 100 μg/mL streptomycin, and maintained in a humidified atmosphere with 5% CO_2_ at 37° C. The shRNA plasmids targeting *LDHA* transcript (target sequence: 5′-CCCAAAAACCGTGTTATTGGAAGC-3′) was obtained using lentiviral vector backbone. The cells were transfected plasmids with Attractene Transfection Reagent (301005, Qiagen, The Netherlands).

### Cell viability assay

Cell viability was examined using MTT assay. Transfected NCI-H441 cells were plated into 96-well plates at a density of 2×10^3^ cells/well. After incubation for 48 h, adding 10 μL MTT (5 mg/mL, Sigma Chemicals Co., USA) per well and incubated for 4 h. After the absorbance values (OD) at 570 nm were measured, 100 μL DMSO per well was added and incubated at 37° C for 10 min in the dark. The cell viability was analyzed according to the OD value.

### Wound healing assay

The cells were plated into 6-well plates at a density of 6 × 10^6^ cells/well, empty vector plasmid and shLDHA plasmid were transfected separately on the next day. Then, the transfected NCI-H441 cells were scraped and the suspension was rinsed with PBS. At 0, 12h and 24h after incubation, the cell migration images were acquired from an inverted microscope.

### Transwell assay

For the migration experiment, 5 × 10^4^ NCI-H441 cells were suspended in 200 μL serum-free medium and seeded in the upper chamber of the transwell. Subsequently, 500 μL RPMI-1640 medium containing 20% serum was added to the lower chamber. For the invasion assay, the Matrigel was diluted with serum-free medium 1:8 before inoculation, then coated the upper chamber and the other steps were followed for the migration assay. After culturing the cells for 48 hours, the cells were fixed and stained by first passing them through the bottom of the lower chamber, and then photographing and counting were completed.

### Statistical analysis

The experiments data were analyzed using GraphPad Prism 8.0 software. The data were presented as the mean ± standard deviation (Mean ± SD) from at least three separate experiments. The student’s t-test was used to analyze paired samples, and the ANOVA analysis was performed to detect unpaired samples. The p < 0.05 was considered to be statistically significant. *p < 0.05, **p< 0.01, ***p< 0.0001.

### Data availability

All data included in this study are available by contacting the corresponding authors.
